# The development of a novel virtual reality simulation module for canine laparoscopic ovariectomy

**DOI:** 10.1186/s13028-025-00815-8

**Published:** 2025-06-02

**Authors:** Jennie Redander, Kerstin Anagrius, Karolina Brunius Enlund, Flemming Bjerrum, Lena Ström, Odd Höglund

**Affiliations:** 1 Department of Clinical Sciences, Faculty of Veterinary Medicine and Animal Science, Swedish University of Agricultural Sciences, Ulls väg 26, Uppsala, 756 51 Sweden; 2 Evidensia Djursjukvård AB, Östhammarsgatan 74, Stockholm, 115 28 Sweden; 3 Gastrounit, Surgical section, Copenhagen University Hospital - Amager and Hvidovre, Kettegård Alle 36, Hvidovre, 2650 Denmark; 4 Copenhagen Academy for Medical Education and Simulation (CAMES), Ryesgade 53B, København, 2100 Denmark; 5 Department of Clinical Medicine, Faculty of Health and Medical Sciences, University of Copenhagen, Blegdamsvej 3B, Copenhagen, 2200 Denmark

**Keywords:** Dog, Education, Endoscopic surgery, Minimally invasive, Training, Veterinary

## Abstract

Canine ovariectomy is a commonly performed procedure in veterinary practice and can be done via an open celiotomy or a laparoscopic approach. Laparoscopy requires different psychomotor skills from those involved in open surgery. Many of these basic skills can initially be acquired through simulation-based training. Surgical simulation training benefits both the surgeon and the patient by offering the acquisition of new skills in a less stressful environment while lowering the risk for the patient. Virtual reality (VR) simulation training is well established in human medicine, but no VR modules have previously been available for veterinary procedure training for laparoscopy. This project aimed to develop a VR simulator module for canine laparoscopic ovariectomy on the simulator LapSim^®^ (Surgical Science, Gothenburg, Sweden). The simulator module could offer veterinary surgeons the opportunity to gain the basic technical skills required for canine laparoscopic ovariectomy. Surgeons with experience in laparoscopic ovariectomy acted as content experts, and the development was a stepwise iterative process. The three-port technique was chosen; it included locating and lifting the uterine horn and sealing and transecting tissue to free the ovaries. The organs and tissues the surgeon interacted with during the simulation were the spleen, intestines, pancreas, ovaries, uterine horns, suspensory ligaments, mesometrium and mesovarium. The module included simulating haemorrhage from the spleen, ovarian and uterine vessels. Suggestions for the continued development of the simulation were identified. Examples were an increased haptic feel in some instances of tissue manipulation and the possibility of introducing a variation in the length of the ovarian pedicle. The last step of the simulated procedure included visual guidance for verification of haemostasis. Even though guidance is not part of a real-life operation, identifying anatomical landmarks was considered valuable from an educational perspective. A validity investigation study has been initiated to evaluate the module as an assessment tool.

**Supplementary Information:**

The online version contains supplementary material available at 10.1186/s13028-025-00815-8.

## Findings

Elective ovariectomy and ovariohysterectomy of a healthy female dog is a routine surgical procedure in veterinary medicine. The surgery can be done through an open abdominal technique, but it is also a procedure where a laparoscopic approach is suitable [1]. The laparoscopic method has several patient benefits compared to the open technique, such as shorter recovery time [2], fewer noxious stimuli [3], reduced analgesia requirements [4], and fewer wound healing complications [5]. However, minimally invasive surgery requires procedure-specific training as the technical skills required differ from those needed for a traditional laparotomy [6]. Simulation-based training offers the possibility to gain basic technical skills, which can be further developed under supervision in the operating room [7]. Training in a simulated environment lessens the risk for the patient by reducing the risk of errors in the operating room [8], and it also increases the trainees’ confidence [9]. Simulation methods available to veterinary surgeons who want to learn laparoscopic surgery include cadavers and box-trainers of varying complexity [10, 11]. For medical doctors, virtual reality (VR) models that simulate procedures based on human anatomy are also accessible. In a recent systematic review evaluating augmented and virtual reality in veterinary medicine [12], there was no mention of any publications reporting on available procedure training on VR simulators for veterinary practitioners. VR simulators have several advantages; they allow for easy repetition during training since they do not require replacing components or live animals, or cadavers. Additionally, they can record standardised data and provide automated feedback on performance. This project aimed to develop a novel VR simulation module of canine laparoscopic ovariectomy for the simulator LapSim^®^ (Surgical Science, Gothenburg, Sweden), offering veterinary surgeons the opportunity to gain basic technical skills through training in a simulated procedure.

**Fig. 1:**
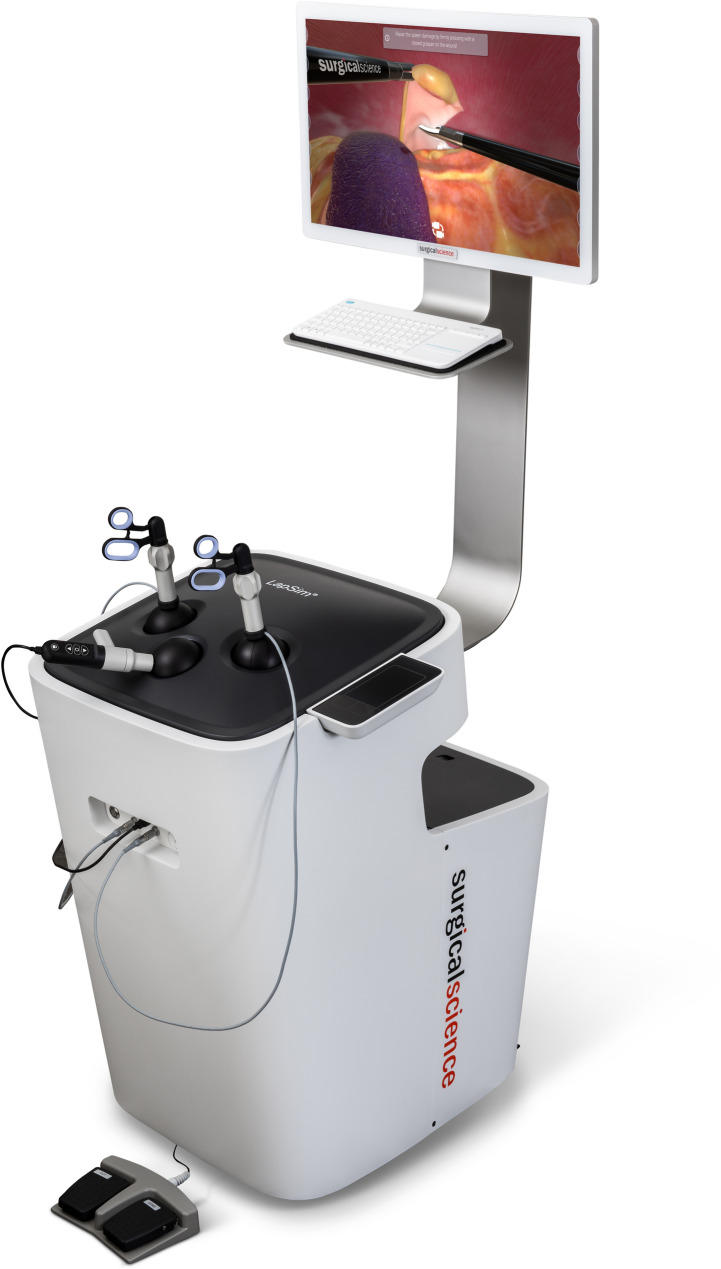
The module Canine ovariectomy was developed for the simulator LapSim^®^ from Surgical Science (Gothenburg, Sweden). Image used with permission by Surgical Science

The canine laparoscopic ovariectomy module was developed in collaboration between Surgical Science (Gothenburg, Sweden) and the Swedish University of Agricultural Sciences (Uppsala, Sweden). The module was created for LapSim^®^ (Fig. 1), a well established VR simulator for assessment and training in human surgery [13]. The simulator has haptic feedback, where the physical sensation of actual contact with the tissue is generated through the instruments during simulation. The simulator continuously records performance data throughout the simulation, such as time, instrument movements, blood loss, errors, etc. After each simulated session, the simulator can provide automated feedback on performance.

During development, a professor of small animal surgery (primary investigator) and a senior veterinary surgeon, both with several years of laparoscopic and teaching experience, acted as content experts. The primary investigator, the funders, and Surgical Science created initial goals for the project that stated what the simulation should contain. The simulator module should represent the canine anatomy correctly, have haptic feedback, and the psychomotor skills included should be relevant to the performance of a canine laparoscopic ovariectomy. The development was a stepwise iterative process between the software developers and the content experts until the product was deemed finished concerning the initial goals.

**Fig. 2:**
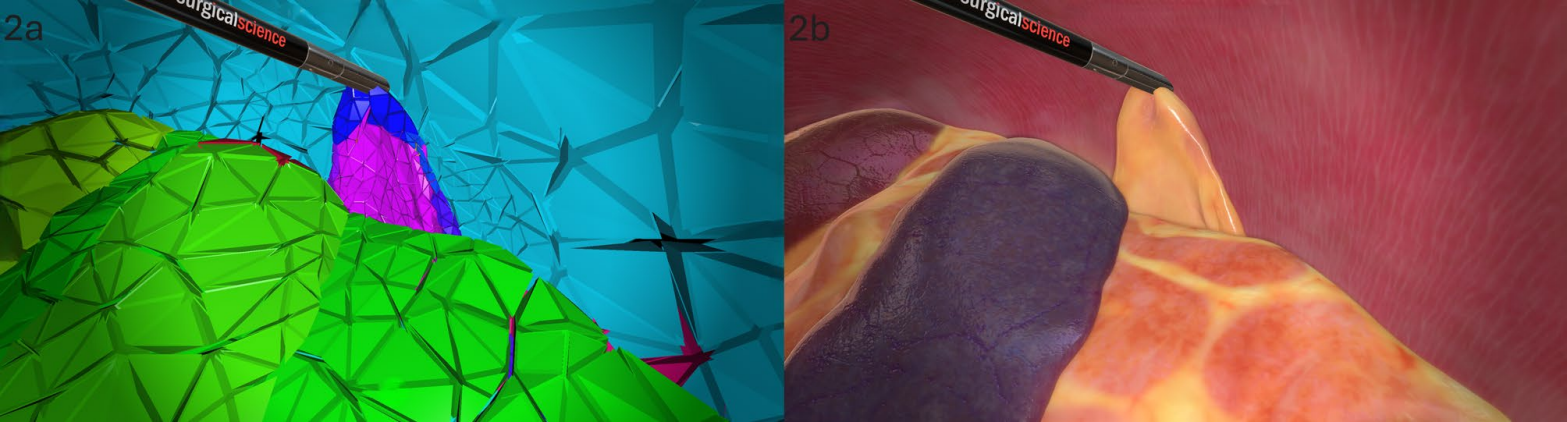
2**a**: Screenshot from the early stage of the Canine ovariectomy module development. 2**b**: Screenshot from a later stage of the Canine ovariectomy module development. Intestines, spleen, liver and a grasper holding the ovary can be seen. Images used with permission by Surgical Science

A computed tomography (CT) scan of the abdomen of a female Australian cattle dog (approximately 20 kilos) provided the foundation for measurements of the simulated abdominal cavity and the size of the included organs. CT imaging was performed before and after the administration of intravenous Iohexol, and it was done for reasons unrelated to this project. The dog was scanned at isotropic resolution 1 mm CT 3D, multiplane reconstruction was performed with 1 mm slice thickness and a B30s kernel of the Spine Helical series (Somatom Definition AS 64; Siemens, The Netherlands). Surgical Science used internal software to translate the CT image into a 3D model. The software developers created the abdominal tissues and organs based on anatomic drawings, photos and videos of several different canine laparoscopic ovariectomy procedures (Fig. 2). This was done using Autodesk Maya (Autodesk, Inc.), a 3D computer graphics application, along with internal software from Surgical Science. To become acquainted with the actual look and feel of the organs, the software developers partook in study visits where they watched live laparoscopic surgeries. Furthermore, they visited the Swedish University of Agricultural Sciences’ pathology department and participated in cadaver exercises alongside veterinary students, which allowed them to palpate organs.

The abdominal wall represented the outer boundary of the simulated cavity; the organs and tissues included were the spleen, intestines, pancreas, ovaries, uterine horns, suspensory ligaments, mesometrium and mesovarium. Vessels were added to the spleen, the ovaries and the uterus. In case of incomplete sealing of the arteries and veins of the ovaries and uterine horns, haemorrhage occurred. Additionally, diffuse bleeding was induced from the spleen in the case of incautious treatment of the organ. In case of haemorrhage from ovarian or uterine vessels, the trainee could stop it by gripping the tissue and sealing it. In case of haemorrhage from the spleen, it could be stopped by the trainee applying 15 s of pressure to the point of bleeding with one of the instruments. Blood accumulated in the abdomen near the ovarian pedicle, reflecting the total amount of blood if haemorrhage occurred.

A three-port technique was chosen for the simulated procedure. The patient was positioned at a pre-set, non-adjustable, slight Trendelenburg and side tilt to improve the visualisation of the organs. The VR simulation included the regular instruments offered by the LapSim simulator. At the beginning of the simulation, a grasping forceps was presented in one hand, and a bipolar vessel sealer/divider device resembling the type LigaSure (Medtronic) was presented in the trainee’s other hand. During the procedure, it was possible to change instruments. Different types of forceps and retractors were available to choose from. There was also a suction tool in case the removal of pooled blood was needed before verifying haemostasis. The simulator controlled the camera and thereby mimicked the procedure of the 3-port technique with an assistant manoeuvring the camera, except at the end of the procedure, where the trainee managed the camera themselves to control for haemostasis. Once the ovaries had been successfully removed, visual rings appeared on the screen to guide the process of verifying haemostasis, completing the simulation.

Several decisions were made during the development process. These decisions were based on the content experts’ knowledge of the procedure, their experience teaching surgery, and the technical and economic feasibility. A three-port technique was chosen: two ports for instruments and one port for the camera [14]. One and two-port techniques are also described [4, 15]. Still, a three-port technique, with a good opportunity of practising bimanual dexterity, was deemed suitable for the initial process of learning laparoscopic ovariectomy. Furthermore, the simulation of placing a transabdominal suspension suture to fixate the proper ligament towards the abdominal wall, as is done in one- and two-port techniques, was deemed too complicated and less suitable for virtual reality simulation. In addition to the procedural steps, one key aspect of the collaboration between content experts and software developers was ensuring that the organs behaved and interacted realistically. Another important area was calibrating the tissues’ density, weight and tactile feel. These adjustments were based on the content experts’ subjective opinions.

Upon the project’s conclusion, potential simulation improvements were identified. There was, for example, a lack of proper fixation of the spleen, which allowed unrealistic anatomical relocation of the organ if it were excessively manipulated. Based on the content experts’ experience, the spleen could also bleed slightly too easily, but it was concluded that this could potentially have pedagogical value, as it might encourage the trainee to handle the spleen carefully. The procedure ended with verifying haemostasis, where visual rings emerged on the screen to guide the trainee. However, this was at the expense of a realistic experience, as it was not authentic compared to the process of live surgery, where there is no external guidance for the necessary steps. Another area of potential improvement was the haptic feedback. There were occasions during the simulation where it was not as pronounced as it would be during a real procedure, for example, when grasping and cutting through the tip of the uterine horn. The simulation was not a complete procedure as port placement was not included, and the simulation ended with haemostasis control, i.e. exteriorisation of ovaries and closing of incisions were omitted. A potential addition to the module could be an optional variation in the length of the ovarian pedicle, since this could offer alternative difficulty levels.

Despite these possible areas of continued development, the module could potentially teach the trainee the essential procedural steps from locating the uterine horn to controlling the haemostasis of the ovarian and uterine vessels. Beyond these procedural steps of a canine ovariectomy, the trainee practices necessary psychomotor skills for laparoscopy, such as depth perception, hand-eye coordination, adaptation to the fulcrum effect and bimanual dexterity.

The virtual reality simulator module for laparoscopic canine ovariectomy was developed as a pedagogical tool to aid veterinary surgeons in gaining technical skills and procedural knowledge. It was made commercially available by Surgical Science to the veterinary community in 2023. A validity investigation study has been initiated to provide the necessary data to evaluate the module’s usefulness as an assessment tool.

## Electronic supplementary material

Below is the link to the electronic supplementary material.

Supplementary Material 1: MP4**-**file. A short video of a part of the simulation Canine ovariectomy on the left side. Video used with permission by Surgical Science

## Data Availability

Not applicable.

## Declarations

### Prior publication

Data have not been published previously.
